# Neuroinflammation and Autophagy in Parkinson’s Disease—Novel Perspectives

**DOI:** 10.3390/ijms232314997

**Published:** 2022-11-30

**Authors:** Danail Minchev, Maria Kazakova, Victoria Sarafian

**Affiliations:** 1 Department of Medical Biology, Medical University-Plovdiv, 4000 Plovdiv, Bulgaria; 2 Research Institute at Medical University-Plovdiv, 4000 Plovdiv, Bulgaria

**Keywords:** Parkinson’s disease (PD), neurodegeneration, neuroinflammation, autophagy

## Abstract

Parkinson’s disease (PD) is the second most prevalent neurodegenerative disorder. It is characterized by the accumulation of α-Synuclein aggregates and the degeneration of dopaminergic neurons in *substantia nigra* in the midbrain. Although the exact mechanisms of neuronal degeneration in PD remain largely elusive, various pathogenic factors, such as α-Synuclein cytotoxicity, mitochondrial dysfunction, oxidative stress, and pro-inflammatory factors, may significantly impair normal neuronal function and promote apoptosis. In this context, neuroinflammation and autophagy have emerged as crucial processes in PD that contribute to neuronal loss and disease development. They are regulated in a complex interconnected manner involving most of the known PD-associated genes. This review summarizes evidence of the implication of neuroinflammation and autophagy in PD and delineates the role of inflammatory factors and autophagy-related proteins in this complex condition. It also illustrates the particular significance of plasma and serum immune markers in PD and their potential to provide a personalized approach to diagnosis and treatment.

## 1. Introduction

Parkinson’s disease (PD) is the second most common neurodegenerative disorder after Alzheimer’s disease. It affects about 1% of the population over the age of 60 [1], with the total number of patients exceeding 6.1 million worldwide. As a highly diverse and complex pathology, PD is represented by a plethora of motor symptoms such as tremor, muscle rigidity, bradykinesia, and postural instability. Non-motor symptoms, including cognitive and behavioral impairments, sleep irregularities, sensory and autonomic dysfunction, are also common in PD [2,3,4]. The key histopathological characteristic associated with this condition is the focal degeneration of dopaminergic neurons, which occurs in *substantia nigra pars compacta* of the midbrain, as well as in *locus ceruleus* and other brain areas. The loss of dopaminergic neurons that project from the *substantia nigra* to the *striatum* causes the primary motor symptoms of PD. As the disease progresses, neurodegeneration affects other brain areas, thus causing the non-motor symptoms of the disease. The neuronal loss co-occurs with the formation of Lewy bodies: cytosolic inclusions of clumped proteins, which are major cytological features of PD [5]. There are several aspects of the etiology and pathogenesis of PD that remain incompletely elucidated. It has been suggested that age, genetic predisposition, and environmental stressors may play a significant role in the onset and the development of this complex disorder. A variety of cellular and molecular characteristics of PD have been repeatedly described in human tissue samples, cell lines, human brain 3D-organoids, and animal models. These include abnormal folding and aggregation of alpha-synuclein (α-Syn), mitochondrial dysfunction, impaired protein degradation (in both ubiquitin-dependent and autophagy-dependent manner), neuroinflammation, and oxidative stress [6]. 

The present review aims to demonstrate the involvement of neuroinflammation and autophagy in PD development and to summarize the role of cellular and soluble inflammatory factors and autophagy-related proteins in this complex condition. Another major objective is to illustrate the specific role of plasma and serum immune markers in PD and their potential to provide a personalized approach to diagnosis and treatment. 

## 2. Neuroinflammation in PD

The inflammatory response serves to efficiently eliminate the causative agent and to facilitate tissue repair [7]. The initiation and progression of inflammation depend on the coordinated interaction between immune and non-immune cells and the fine regulation of inflammatory mediators. Primary inflammatory stimuli (molecules and structures of microbial origin, aggregated or misfolded proteins) and cytokines—interleukin-1β (IL-1β), interleukin-6 (IL-6), and tumor necrosis factor-α (TNF-α)—promote inflammation. It is triggered as a result of interaction with the Toll-like receptors (TLRs), IL-1 receptor (IL-1R), IL-6 receptor (IL-6R), and the TNF receptor (TNFR) [8]. The activated receptors activate intracellular signal transduction cascades, including the mitogen-activated protein kinase (MAPK), nuclear factor kappa-B (NF-κB), and Janus kinase signal transducer and activator of transcription (JAK-STAT) pathways. Effector macrophages and lymphocytes release pro- and anti-inflammatory cytokines that recruit other leucocytes and modulate the inflammation itself via a complex network of interactions, thus regulating both the expansion and the intensity of the process [9]. 

The inflammatory response in the CNS (neuroinflammation) has been directly associated with viral and bacterial diseases, autoimmune and neurodegenerative conditions, trauma, vascular damage, and neuropsychiatric disorders. Neuroinflammation can in-crease the neuronal excitability, trigger cellular damages, and augment the permeability of the blood-brain barrier [10]. 

Different studies have demonstrated that neuroinflammation participates not only in typically inflammatory diseases such as viral encephalitis but also in neurodegenerative conditions, including PD [11,12]. Neuroinflammation in PD involves activation of microglia and T-lymphocytes alongside an increased expression of pro-inflammatory cytokines. Experiments with animal models of PD have indicated that neuroinflammation is profoundly involved in neuronal cell death, despite not being its primary cause. In agreement with this assumption, available evidence suggests a significant role of glucocorticoid receptors in modulating microglial reactivity and their substantial dysregulation in the inflammation-mediated neuronal degeneration [13].

### 2.1. Microglia in PD

Microglial cells are the resident macrophages of the brain [14]. First discovered by Pío del Río Hortega [15], they serve as primary cells of innate immunity in the CNS and play a crucial role in maintaining the homeostasis of the brain [16]. Microglia participate in synaptogenesis, synaptic pruning, neural progenitor-cell growth and differentiation, and myelinogenesis [17,18,19]. Microglial activation is a complex response against infection or injury that produces two functionally distinct phenotypes: M1 and M2 [20]. According to the general model, although greatly simplified, M1 microglia secrete pro-inflammatory cytokines (IL-1β, IL-6, IL-12, TNFα) that stimulate neurodegeneration [21]. These mediators broaden the immune response and may directly contribute to neuronal death. TNFα is known for its pro-apoptotic activity which, in neurons, depends on the downregulation of c-Rel, a NF-κB homologue that inhibits cell death and promotes neuronal survival [22]. M1 cells also upregulate enzymes that produce reactive oxygen species with antimicrobial function, thus elevating oxidative stress. Simultaneously, microglial metabolism shifts from oxidative phosphorylation (OXPHOS) to glycolysis, allowing microglia to adapt to increased energy demands. Metabolic reprogramming leads to faster, although less efficient, production of ATP for proliferation, cytokine production, and ROS generation [23,24]. It has been demonstrated that the glycolytic inhibitor deoxy-D-glucose (2-DG) decreases TNFα and IL-6 in microglia through NF-κB suppression, inducing microglial death [25]. Furthermore, an in vitro study on BV-2 microglial cells revealed an elevated lactate production and decreased mitochondrial activity following lipopolysaccharide stimulation [26]. In contrast with M1, M2 cells express factors involved in the inhibition of inflammation and promotion of tissue repair. They secrete substances, such as IL-10, to reduce the activity of pro-inflammatory cells. M2 microglia also express high levels of phagocytic receptors to promote the clearance of cell debris [27]. Nevertheless, high-throughput studies have revealed that microglial heterogeneity is even more complex, suggesting the presence of a wider spectrum of microglial phenotypes [28]. To date, little is known about the molecular mechanisms of microglial heterogeneity. 

Studies of 1-methyl-4-phenyl-1,2,3,6-tetrahydropyridine (MPTP)-induced PD mice models have demonstrated that microglial activation is a prominent and persistent feature of PD [29,30,31]. Even the fact that *substantia nigra* constitutes the predominantly affected site in PD is in concordance with the higher abundance of microglial cells in this brain region [32]. Microglia exert versatile roles in neuroinflammation, serving as both a damaging and a protective factor. When activated, microglial cells infiltrate the site of neuroinflammation, where they perform phagocytosis and secrete both pro- and anti-inflammatory cytokines [33]. The cytokine synthesis and secretion are hallmarks of microglial activation as part of the early inflammatory response and persist throughout the disease progression [34,35,36]. Secretion of pro-inflammatory factors such as IL-1β, IL-12, TNFα, and inducible nitric oxide synthase (iNOS) greatly stimulates neuroinflammation and often corresponds to significant neuronal loss. Conversely, the production of anti-inflammatory cytokines such as IL-4, IL-10, IL-13, TGFβ, and IGF-1 by microglial cells suppresses inflammation and promotes neuroprotection [33]. From this perspective, a prominent factor that may contribute to microglial activation is the release of α-Syn. It is an abundant neuronal protein that localizes to the presynaptic terminals in the CNS where it regulates vesicular release [37,38,39]. Its native conformation is largely unfolded but the same protein can also exist in abnormal aggregated forms such as oligomers, protofibrils, and fibrils [40]. α-Syn is the main component of Lewy bodies and, as such, significantly contributes to the pathogenesis of PD. In the PD brain, α-Syn is often released by neurons in the extracellular interstitium, which allows its laboratory detection in the bodily fluids of PD patients. Subsequently, the α-Syn-induced microglial activation triggers rapid α-Syn phagocytosis. In this process, the activated microglial cells engage their FcγR receptors in the α-Syn uptake and initiate a sequence of pro-inflammatory events such as NF-κB/p65 translocation and increased secretion of cytokines. These neuroinflammatory effects then result in neuronal loss and chronic neurodegeneration in PD. In addition to α-Syn, other PD risk factors such as DJ-1 and LRRK2 can also participate in the regulation of microglia-mediated inflammation. For instance, LRRK2 deficiency represses inflammation by inhibiting the p38 MAPK and NF-κB pathways [33].

### 2.2. Astroglia in PD

Astroglia constitute the largest population of glial cells in the brain and perform functions essential for the normal physiology of the CNS. Astrocytes mechanically support the neurons and the adjacent capillaries. They maintain the integrity of the blood–brain barrier and its permeability [16,41,42]. Astroglia synthesize and secrete a plethora of neurotrophic factors, including glial cell line-derived neurotrophic factor (GDNF), brain-derived neurotrophic factor (BDNF), nerve growth factor (NGF), and cerebral dopamine neurotrophic factor (CDNF). These neurogenic molecules stimulate and fine tune neuronal development, survival, and plasticity. In addition, CDNF provides neuroprotection and promotes the recovery of damaged dopaminergic neurons [43,44,45,46]. In the structure of the tripartite synapse, astrocytes surround the synaptic cleft where they interact with the pre-and post-synaptic neurons and uptake excessive glutamate [42]. Astrocytes also provide metabolic support for the neurons by transferring lactate for the Krebs cycle. They are able to produce antioxidants and to neutralize neuronal waste products, including aggregated α-Syn and damaged mitochondria [47,48]. Finally, astrocytes are responsible for the remodeling of the nervous tissue by filling the gaps left after neuronal death, forming the so-called astroglial scar [49].

Similar to microglia, astrocytes exist in different functional states. The A1 astrocytic population produces pro-inflammatory factors such as IL-1α, C1q, and TNFα, thus enhancing neuronal death and inflammation. Conversely, the A2 population promotes neuronal survival and neuroprotection after injury [50]. Liddelow et al. (2017) have determined that microglia cause astrocytic activation by secreting the cytokines IL-1α, TNF, and C1q [49]. The authors also demonstrated an elevated production of pro-inflammatory cytokines such as TNF-α, IL-1α, and IL-1β in A1 astrocytes as a consequence of this activation. In their pro-inflammatory state, astrocytes no longer assist neuronal survival but induce cell death by releasing neurotoxic molecules. In turn, astrocytes can modulate microglial activation and microglia-mediated inflammation [50].

Altered astrocytic function is involved in different mechanisms of PD development, such as α-Syn accumulation, neuroinflammation, impaired mitochondrial metabolism and oxidative stress. Of particular interest is the fact that at least eight out of 17 genes of known causative importance for PD are expressed in astrocytes [51]. One of them, PARK7, is even more prominent in astroglia than in neurons and shows noticeable upregulation in astrocytes from PD individuals. The product of this gene, DJ-1, is involved in oxidative stress response, glutamate uptake, and neuroprotection [52].

It has been shown that microglia and astrocytes can exhibit a protective effect on neurons by eliminating extracellular α-Syn. Glial cells engulf and degrade complexes of aggregated α-Syn via proteasomal and autophagic mechanisms [47,48]. Exchange of intracellular materials including α-Syn and intact mitochondria occurs not only between astrocytes and neurons but between neurons themselves [53]. Although α-Syn is predominantly expressed and accumulated in neurons, different studies have reported that α-synuclein aggregates in astrocytes as well. Accumulation of α-Syn can disrupt astrocyte function and accelerate neurodegeneration through mitochondrial dysfunction and impaired autophagy [54].

**Figure 1:**
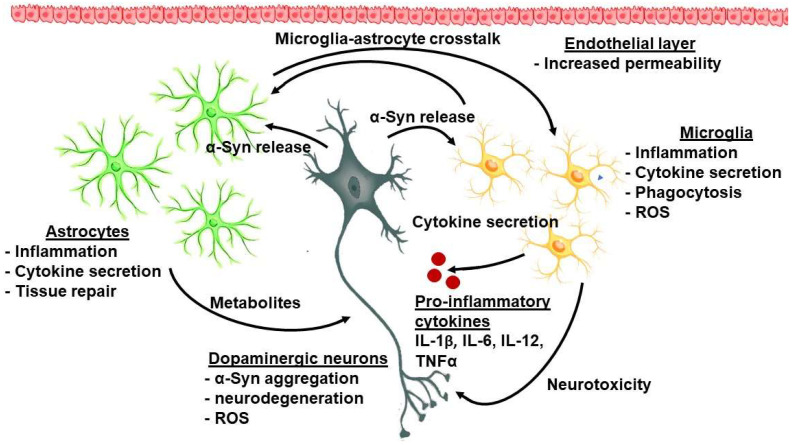
The complex interplay between cellular and molecular factors in PD-related neuroinflammation.

Sonninen et al. (2020) have demonstrated that metabolic changes occur in iPSC-derived astrocytes from PD patients carrying mutant variants of the LRRK2 gene. These astrocytes were characterized by abnormal α-Syn expression, metabolic alterations, impaired Ca^2+^ regulation, and elevated cytokine production [55]. It has been proposed that mitochondrial dysfunction in astrocytes possibly evokes neuronal toxicity by altering the normal glutamate uptake and degradation, Ca^2+^-induced cell death, impaired metabolism, and accumulation of ROS and toxic fatty acids [56]. The interplay between astroglia and microglia is presented in Figure 1.

### 2.3. Inflammatory Cytokines

Numerous reports have revealed the significant association between PD severity and the level of immune markers in plasma and serum.

Notably higher serum levels of the proinflammatory cytokine IL-1β are detected in patients with early PD [57]. However, no significant correlation was found between the IL-1β levels and the clinical scales for PD assessment. Selikhova et al. (2002) described elevated IL-6 in the plasma of patients with early idiopathic PD [58]. Higher abundance of the pro-inflammatory cytokines IL-1β, INF-γ, and TNF-α was detected in PBMCs isolated from PD patients [59]. The levels of TNF-α (but not those of IL-1β and IL-10) correlate with cognition and other non-motor symptoms of PD [60].

It is noteworthy that, contrary to these findings, a significant downregulation of inflammatory cytokines has also been demonstrated in patients with PD. In a study by Rocha et al. (2018), the PD-patient cohort exhibited a lower percentage of T-lymphocytes, including activated T-lymphocytes, in comparison with healthy controls [61]. In accordance with these findings, the authors also described decreased plasma levels of IL-4, IL-6, IL-10, TNF, IFN-γ, and IL-17A in the PD group [61]. In a previous paper, the same authors also detected significantly elevated levels of the soluble TNF-α receptors, sTNFR1 and sTNFR2, in plasma of PD patients, suggesting the inflammatory etiology of PD [62]. Another comparative analysis [63] suggested that IL-6 was significantly higher in patients with PD than in healthy controls. Conversely, the authors found no significant differences in the levels of CRP, sIL-2R, or TNF-α between the two studied groups. Dufek et al. (2008) investigated a panel of inflammatory markers in serum samples from 29 patients with PD and found significant overexpression of only TNF-α [64]. None of the other markers of interest (IL-6, acute phase proteins, and factors of the complement system) showed any abnormal changes in the PD group. There were also no significant correlations between the patients’ clinical state and the levels of the examined serum markers. 

Another cohort study revealed significantly lower serum levels of IL-1α and IL-6 in PD patients than in their age-matched controls [65]. Conversely, the serum IL-1β levels in the PD group appeared significantly higher than those in the control one. Again, the authors observed no correlation between the studied markers and disease severity.

In a study of 83 PD patients and 83 healthy subjects, higher serum levels of TNF-α and lower levels of IL-27 were detected in patients with PD compared to healthy controls (*p* < 0.0001) [66].

All these studies are greatly limited due to the relatively small number of participants. The first large-cohort research to evaluate serum cytokine markers in the context of PD examined 262 newly diagnosed PD patients and 99 healthy controls [67]. It demonstrated that a panel of cytokines is robustly associated with cognitive and motor features of PD. The experimental results revealed higher levels of TNF-α, IL-1β, IL-2, and IL-10 in PD versus healthy individuals. Based on their data, the authors suggest that a more pro-inflammatory profile is associated with impaired cognition and rapid motor regression, while a more anti-inflammatory profile is related to improved cognitive abilities and preserved motor function. Earlier research investigating the role of CRP in 375 PD individuals has suggested that CRP associates with faster motor decline. 

The immense scientific work in clarifying the complex interplay between immunity and PD development delineates the potential usefulness of cytokines as biomarkers of inflammation and neurodegeneration. Probably the most promising future insights will combine clinical data, cellular and molecular features.

### 2.4. YKL-40 in PD-Related Neuroinflammation

The YKL-40 glycoprotein has been established as a prospective biomarker of neuroinflammation in neurodegenerative diseases. YKL-40 has also been debated as a biomarker in diverse medical conditions, including toxoplasmosis [68], autoimmune disorders [69] and hemodialysis inflammation [70]. This protein serves as an acute-phase factor, secreted by a variety of immune cells (especially macrophages) in response to pro-inflammatory signals including IL-1β, IL-6 and IFNγ, and TNFα. It is noteworthy to mention that YKL-40 has a number of different cellular sources (chondrocytes, fibroblast-like synovial cells, vascular smooth muscle cells, and macrophages) [71,72]. Our previous results showed correlation of YKL-40 and neuron-specific enolase levels with clinical scores for assessment of severity and outcome of traumatic brain injury [73]. We proposed that YKL-40 might reflect certain aspects of the response to brain injury, such as neuroinflammation and brain damage. A series of studies have suggested that the levels of YKL-40 correlate with the glial activation and the number of cells involved in neurodegeneration [74,75,76]. Its levels in cerebrospinal fluid (CSF) have been correlated with the disease phenotype of Parkinson’s-related disorders. For instance, Magdalinu et al. (2015) discovered that the levels of YKL-40 were lower in patients with PD compared to those with atypical Parkinson’s syndrome, but still higher than the levels in the control group [74]. No correlations with disease stages or severity were observed in this study. However, the expression data regarding YKL-40 in PD remain controversial. Substantially higher YKL-40 levels in PD patients have also been reported. A two-year follow-up study revealed a significant increase in the concentration of YKL-40 in the CSF of PD patients compared to the baseline levels. Furthermore, the steady increase in YKL-40 levels correlated with the deterioration of cognitive abilities [75]. Conversely, according to other authors, the levels of YKL-40 were lower in patients with PD than in healthy controls or those with multisystem atrophy, progressive supranuclear palsy and corticobasal degeneration. Additionally, the concentration of YKL-40 in CSF appeared lower in degenerative disorders known as synucleinopathies than in tauopathies [76]. In that study, Olsson et al. (2013) evaluated the levels of YKL-40 together with those of the soluble CD14 as markers for astrocyte and microglial activation. They examined CNF and serum samples from 37 controls, 50 PD patients, and 79 P+ patients (with progressive supranuclear palsy, corticobasal degeneration, and multiple system atrophy). The experimental results identified significantly lower YKL-40 levels in the CNF of PD patients compared to healthy controls or participants with multiple system atrophy and tauopathies. A more recent study has reported elevated YKL-40 levels in cerebrospinal fluid (CSF) from patients with AD dementia, but not in those with PD and Lewy body dementia (LBD), in comparison with non-dementia controls. The authors also investigated the possible association between YKL-40 dysregulation in CSF and other inflammatory-markers. They found no relationship between YKL-40 and levels of the astrocytic marker glial-fibrillary acidic protein (GFAP), interleukin-8 (IL-8), monocyte chemoattractant protein-1 (MCP-1), and interferon gamma-induced protein 10 (IP-10) [77]. Additionally, the plasma levels of YKL-40 have been evaluated in the extended spectrum of neurodegenerative dementias. Villar-Piqué et al. (2019) described significantly higher plasma YKL-40 levels in Creutzfeldt-Jakob disease (CJD) with a moderate potential to discriminate CJD cases from controls. Additionally, YKL-40 levels were strongly associated with age but not with gender. In CJD, YKL-40 concentrations appear significantly higher at late disease stages [78]. 

Based on these vast experimental data, the protein YKL-40 may have a potential role as a promising biomarker that reflects the severity of inflammation in PD.

## 3. Protein Factors and Autophagy in PD

Autophagy is an essential catabolic mechanism in which cells degrade misfolded proteins and larger cellular complexes such as excessive organelles. In the process, cytoplasmic components undergo degradation in the lysosomes, releasing molecular building blocks for reuse [79]. This mechanism is crucial for cell homeostasis. Dysfunctional autophagy has been associated with several diseases including malignancies, bone diseases, as well as cardiac and neurodegenerative conditions [80,81,82].

There are three major types of autophagy, which differ in the ways of delivering substrates to the lysosomes. These include microautophagy, chaperone-mediated autophagy (CMA), and macroautophagy [83]. In microautophagy, cellular molecules and complexes are directly engulfed into lysosomes where they are subsequently degraded [84]. In turn, CMA relies on specific recognition of KFERQ-resembling motifs within the target proteins. These motifs interact with the heat shock cognate protein of 71 kDa (HSC70). HSC70 then promotes translocation of the protein substrates into the lysosomal lumen through the lysosome-associated membrane protein 2 receptor (LAMP2A) for degradation. CMA is involved in the clearance of damaged and clumped proteins, and its dysfunction has been associated with neurodegenerative diseases [85,86,87]. It is well-known that HSC70 prevents the clustering of α-Syn into fibers, since it binds with high affinity to the soluble α-Syn. It has been also demonstrated that HSC70 interacts with α-Syn fibers and promotes survival in mammalian cultured cells by reducing the toxicity of α-Syn fibers [88].

Macroautophagy (or autophagy in the narrow sense of the term) is the best characterized type out of the three. The process was initially described by Christian De Duve [89]. In recent years, Yoshinori Ohsumi elucidated the molecular mechanisms of macroautophagy and the genes involved in it [90]. The substrates are encapsulated in double-layer membrane vesicles called autophagosomes that subsequently fuse with the lysosomes to produce hybrid organelles: autolysosomes. Autophagy is a normal physiological process that occurs in healthy cells. However, it is greatly induced under stress conditions such as starvation, reactive oxygen species, and infections that significantly increase the number of autophagosomes [91].

The initiation of autophagy in mammalian cells requires the presence of several protein factors and complexes. The Unc51-like kinase 1 complex (ULK1) consists of a catalytic subunit ULK1, an adaptor subunit ATG13, ATG101, and a FIP200 (focal adhesion kinase family interacting protein of 200 kD). ULK1 is a promising therapeutic target in PD [92]. Activation of ULK1-induced autophagy exhibits a significant protective effect against MPTP-induced motor dysfunction and dopaminergic neurodegeneration in mouse models [93]. The activity of the complex is modulated by phosphorylation. In general, the complex is inactivated via phosphorylation by the mTORC1 (mammalian target or rapamycin, also referred to as mechanistic target of rapamycin/ Complex 1) that suppresses the activity of ATG13 as well. The ULK1 complex is activated by AMPK (adenosine monophosphate-activated protein kinase), which also inhibits mTORC1 by phosphorylating it directly [94,95]. 

Experiments involving knockout and knockdown mouse models have demonstrated the importance of each of the above-mentioned proteins as key markers of autophagy. Knockout mice of ATG13 and RB1CC1 die during embryonic development, while null alleles of either ULK1 or ULK2 express a rather mild effect on autophagy. Conversely, ULK1/2 double-knockout mice die shortly after birth. Fibroblasts isolated from these ULK1/2 double-knockouts are responsive to glucose starvation but show no autophagy induction in response to amino acid deprivation [96,97,98,99,100]. The PI3K/Akt/mTORC1 axis serves as a central negative regulator of autophagy [101,102]. Since mTORC1 suppresses the key autophagy-related complex ULK1 [103], the inactivation of mTORC1 promotes autophagy initiation [104]. In the living cell, such mTORC1 inactivation is often provoked by amino acid scarcity or low insulin levels [104]. The inactivation of mTORC1 mobilizes a class III PI3K complex to complete the early stages of phagophore formation. The activity of ULK1 is tightly regulated by upstream kinases including mTOR, AMPK, and AKT1. Each separate subunit of ULK1 has been extensively studied, but the significance of every single protein–protein interaction within the complex is yet to be elucidated [105,106,107,108]. Most studies report that the ULK1 subunit ATG13 is required for the recruitment of the other subunits of the core complex (ULK1, RB1CC1, and ATG101) and their proper assembly. It has been also reported that ATG13 directly interacts with both phospholipids and proteins. From that perspective, targeting the protein–protein interactions involving ATG13 may provide promising strategies for the modulation of autophagy signaling [109,110,111]. Blocking the interaction between ATG101 and ATG13 exhibits the strongest inhibitory effect on autophagy, while the obstruction of ATG13—ULK1 or ATG13—RB1CC1 interactions shows only limited effects. Additionally, the effects on autophagy resulting from suppression of the ATG13 binding to phospholipids or Atg8 proteins are mild as well. Together, these experimental results suggest that the ATG13—ATG101 interaction may have a significant role as a prospective target in autophagy-related pathologies [112]. Numerous studies involving PD models (including rodents, invertebrates, and cultured cells) have revealed that AMPK activation may exhibit vast neuroprotective effects. AMPK-mediated phosphorylation increases ULK1 activity and induces autophagy, while mTORC1 phosphorylation prevents ULK1 from binding to AMPK. Conversely, mTORC1 inhibition stimulates ULK1 which initiates the autophagosomal formation [113,114]. 

The mTORC1 inhibition stimulates a class III PI3K complex to promote phagophore formation. The class III PI3K complex consists of several proteins including vacuolar protein sorting (VPS) 34, VPS15, Beclin-1, and Atg14 that drive the early stages of phagophore growth. The VPS34 subunit of the PI3K complex produces phosphatidylinositol-3-phosphate (PI3P) simultaneously with the formation of omegasomes [115]. It has been shown that experimentally induced overexpression of Beclin 1 can decrease the accumulation of α-Syn aggregates and reduce the related cell damage. Moreover, the transfer of a lentivirus vector (LV) expressing the Beclin 1 cDNA into a mouse model restores the expression of the presynaptic marker synaptophysin and the postsynaptic marker MAP2. In addition to the reduction of α-Syn accumulation, Beclin 1 overexpression promotes autophagy induction as demonstrated by the elevated levels of LC3. In line with this, the reduced accumulation of α-syn and the elevated autophagy is also confirmed by an ultrastructural analysis [116]. In vitro studies have suggested that the absence of Vps34 results in disrupted autophagy. According to Jaber et al., such absence affects predominantly the late autophagosome formation and leaves the early stages of autophagy largely unaffected. Additionally, it has been also demonstrated that Vps34 plays a central role in amino acid-induced mTOR signaling [117]. The function of Vps15 in autophagic clearance of aggregate-prone proteins has been clearly confirmed. Since increased autophagy can ameliorate aggregate-evoked cell death, targeted modulation of the Vps34-Vps15 complex may open perspectives for the development of more precise therapeutic strategies against neurodegenerative pathologies [118]. Silencing of Atg14 in HeLa cells abolishes autophagosome formation almost completely. Moreover, electron microscopic imaging has shown that autophagosomes are virtually absent in Atg14 knockdown cells. These observations suggest that Atg14 participates in autophagosome formation rather than in autophagosome maturation [119].

Following omegasome formation, the PI3P-effector protein WIPI (WD repeat domain, phosphoinositide-interacting protein) recruits Atg2 to the phagophore. Atg2 serves to establish a connection between the phagophore and the endoplasmic reticulum, and to transfer lipids to the growing autophagosome membrane [120]. LC3, a ubiquitin-like protein, is engaged in the phagophore elongation and closure via binding to phosphatidylethanolamine. In turn, another ubiquitin-like protein, Atg12, binds covalently to Atg5 through Atg7 and Atg10. The Atg12-Atg5-conjugate then binds to Atg16L1, which allows the conjugation of LC3 to phosphatidylethanolamine [121,122]. Additionally, several studies have shown that decreased expression of Atg5 or Atg7 in knockout mice leads to neuronal loss and protein accumulation in the remaining neurons [123,124]. 

The lipidized LC3 shows E3-like activity, which allows the selective degradation of specific substrates [125,126]. Furthermore, it has been determined that LC3 overexpression has neuroprotective significance and enhances autophagic clearance of Aβ clearance in vitro and in vivo [127]. Conversely, impaired LC3 lipidation causes accumulation of unsealed phagophores, which suggests a role of LC3-phosphatidylethanolamine interaction in autophagosome biogenesis [128]. Intriguingly, functional autophagosomes have been reported to develop even in the absence of all ATG8 factors (one of which is LC3) [129]. Another functional defect that also causes accumulation of unclosed autophagosomes is the knockdown of ATG2, a factor that exerts a crucial role in the lipid transfer towards the growing phagophore [130]. Disrupted protein homeostasis associated with accumulation of α-Syn microaggregates in dopaminergic neurons is a constantly observed hallmark of PD. These microaggregates allow the dissemination of α-synuclein among closely associated neurons, although the exact mechanism is yet to be revealed [131]. Stykel et al. (2021) have demonstrated that mutant forms of α-syn (specifically A53T and E46K) engage functional LC3B monomers into insoluble microaggregates. Moreover, the authors also discovered that constitutive inactivation of LC3B enhances α-syn accumulation, while LC3B activation inhibits clumping and facilitates α-syn clearance [131].

The formation of the autophagosome completes when the extending ends of the phagophore fuse with one another with the assistance of ESCRT (endosomal sorting complex required for transport). At least two different types of SNARE proteins—SNAP29 (synaptosomal-associated protein 29) and lysosomal VAMP7/VAMP8 (vesicle-associated membrane protein)—mediate the fusion of autophagosomes and lysosomes. The merging of autophagosomes with lysosomes produces autolysosomes, in which the engulfed materials are decomposed [132,133].

Abnormal levels of α-Syn disrupt the optimal autophagy turnover by impairing the merging of phagosomes with lysosomes. α-Syn also stimulates the release of extracellular vesicles and increases the presence of autophagy-associated proteins in those vesicles. The observed effects result from the capability of α-Syn to decrease the levels of SNAP29, one of the key factors mediating autophagosome–lysosome fusion. In general, α-Syn overexpression causes a reduction in SNAP29 levels, which impairs the incorporation between autophagosomes and lysosomes. That results in fewer autolysosomes being formed, which impairs the degradation of cellular cargoes. As a compensatory mechanism, autophagosomes merge more readily with the cell surface membrane to release vesicles into the extracellular space. Accordingly, the effects of a SNAP29 knockdown closely resemble the α-Syn impact on autophagy while SNAP29 co-expression ameliorates the α-Syn-induced alterations on autophagy turnover and vesicle release and reduces neuronal death [134]. 

Taken together, these observations support the concept that abnormalities in the autophagy pathway play a central role in the pathogenesis of PD. It is also assumed that key factors of autophagy may serve as potential therapeutic targets for PD.

Autophagy pathways in the CNS that involve PI3K, Akt and mTOR offer attractive perspectives for better understanding of neurodegeneration and the development of novel therapeutic strategies. PI3K, Akt and mTOR are essential regulatory factors that determine cell fate, not only during PD but also in the course of other disorders, such as HD, AD, epilepsy, and traumatic injury. However, the delicate interplay between these factors and the extent to which they can regulate autophagy may yield unanticipated practical outcomes. Further efforts are still needed for the complete elucidation of the finer regulatory mechanisms of autophagy, which will allow the development of precise clinical strategies [135].

Even though autophagy may be initiated at a certain disease stage, chronic inhibition of autophagy is a pivotal pathological characteristic of PD. Reduced autophagic capacity is consistent with the accumulation of abnormal proteins and protein aggregates as well as aging and dysfunctional organelles, which accelerate cell degeneration. Given the crucial role of autophagy in PD, investigators are in search of small molecules that modulate autophagy and may potentially improve the symptoms of the disease. Prospective candidate compounds that have displayed neuroprotective effects in experimental PD models are rapamycin, trehalose, and lysosome modulators [135].

Using structure-based drug design Ouyang et al. (2018) discovered a compound with the designated name 33i (BL-918) that induces autophagy by activating the ULK1 complex. The autophagy-promoting effects were confirmed experimentally in an in vitro assay with the use of SH-SY5Y cells. The 33i compound demonstrates its protective effect on SH-SY5Y cells even following an MPP treatment. Moreover, the activator protects against MPTP-induced movement impairment and death of dopaminergic neurons by enhancing ULK1-mediated autophagy in mouse models of PD. Taken together, these results suggest the therapeutic potential of 33i as a prospective drug for PD [93]. 

It has been demonstrated that resveratrol treatment alleviates 6-OHDA-provoked degeneration of dopaminergic neurons and decreases pro-inflammatory cytokines in rats [136]. Furthermore, in MPTP mice, resveratrol reduces depletion of dopamine and tyrosine hydroxylase in the striatum and loss of dopaminergic neurons in the *substantia nigra* [137]. Resveratrol’s cytoprotective effects are greatly diminished by inhibiting AMPK with compound C [138]. Furthermore, resveratrol decreases the levels of α-Syn in PC12 cells overexpressing it. This effect is prevented by Beclin-1 knockdown or lysosomal inhibition, suggesting that macroautophagy is crucial for resveratrol-induced clearance of α-Syn [139]. 

GPA exerts neuroprotection in MPTP-treated mice and prolongs the lifespan in Drosophila by increasing AMPK activity [140]. Consumption of 1% GPA with food for 4 weeks significantly enhances both AMPK activity and mitochondrial respiration in the striatum. It ameliorates the MPTP-related degeneration of dopaminergic neurons in the *substantia nigra* of experimental models [141]. 

Another widely tested drug, metformin, significantly reduces the MPTP-triggered neurodegeneration and dopamine decline in mice and reinstates normal motor function during a rotarod test [142]. Additionally, metformin increases macroautophagy and decreases α-Syn, reactive microglia, and the levels of pro-inflammatory cytokines. These conclusions were further confirmed in SH-SY5Y cells treated with MPP+; these showed improved survival capabilities and reduced release of lactate dehydrogenase (LDH) following metformin administration. Metformin also increases macroautophagy-related markers, while decreasing ROS levels and the number of dysfunctional mitochondria. Conversely, these protective effects are nullified by the application of compound C (a potent AMPK inhibitor) and the macroautophagy inhibitor 3-Methyladenine (3-MA). [142] These results indicate that metformin-mediated AMPK activation exerts significant protective effects through a plethora of mechanisms, such as increased α-Syn clearance, improved mitochondrial fitness, and reduced neuroinflammation. In another experiment involving murine models, metformin significantly elevated the expression of superoxide dismutase and catalase, thus reducing the oxidative stress in the studied animals [143]. Metformin also increased expression of the neurotrophic factor BDNF in MPTP mice undergoing a 21-day treatment. Inhibition of the TrkB neurotrophin receptor in SH-SY5Y cells treated with MPP+ cancels the neuroprotective effects of metformin, suggesting that the proper BDNF signaling may play a critical role in the mechanisms of metformin treatment [144]. High expression of the stress-inducible protein Sestrin-2 stimulates macroautophagy and cell survival and decreases α-Syn accumulation in MES 23.5 cells treated with rotenone. These cytoprotective effects appear to depend on AMPK, since metformin can rescue the impact of a Sestrin-2 knockdown on macroautophagy [145]. In a separate study, mice with Clk1 deficiency and impaired AMPK function demonstrated decreased autophagy and increased susceptibility to MPTP-triggered neurodegeneration. In these mice, metformin restores macroautophagy and reduces the MPTP-related motor symptoms and loss of dopaminergic neurons, further suggesting the role of AMPK-mediated macroautophagy in PD [146]. 

Neurotrophins have been established as important modulators of autophagy in neurons. Available data suggest that neurotrophins including BDNF support neuronal survival, at least partially, by modulating autophagy. It has been shown that in neurons, BDNF/TrkB signaling assists cell survival by modulating the PI3K/Akt/mTOR and autophagy. It is noteworthy to mention that BDNF-related effects on autophagy depend on the nutritional state of the neuron and its localization in a particular brain region. For instance, in cultured cortical neurons under hypoxic conditions, BDNF enhances cell survival by inhibiting the PI3K/Akt/mTOR pathway and, thus, enhancing autophagy. Conversely, in cell cultures of hippocampal neurons under no metabolic challenge, BDNF suppresses autophagy again via the PI3K/Akt/mTOR pathway. Additionally, studies involving animal models have demonstrated that conditional deletion of BDNF in the neural lineage results in increased LC3 and decreased p62 levels in the brain, which further confirms the role of BDNF in autophagy modulation [147,148,149,150].

A series of studies of transgenic mice, and animal and cell models of PD have underlined the role of PD-related protein factors, mostly α-Syn and LRRK2, in the processes of autophagy [151,152]. Of particular importance is the fact that autophagy is the only mechanism used by eukaryotic cells to break down clumped proteins and damaged organelles that cannot be digested by the proteasome. α-Syn molecules with native folding are degraded mainly via chaperon-mediated autophagy involving HSP70 and LAMP2A. However, mutant α-Syn variants are resistant to such degradation. As a result, they aggregate in the cytosol, thus causing significant neuronal damage. The cell then engages macroautophagy, in which the α-Syn clumps become enclosed in endophagosomes [153]. Accumulation of autophagosomes has been found in brain samples from patients with PD, which confirms the possible induction of autophagy. According to Dehay et al. (2010), the accumulation of autophagosomes and the loss of dopaminergic neurons result from a significant decrease in the number of lysosomes in these neurons [154]. The authors argue that levels of LAMP-1 as a marker for the lysosomal dynamics in mouse models of PD are characterized by a significant decrease during the course of the pathological process. Lower levels of LAMP-2A have been recently described in samples from the cortex and hippocampus of PD patients [155]. In the same way, the immunoreactivity of LAMP-1, cathepsin D (CatD), and HSP73 has proven to be significantly lower in the neurons of *substantia nigra* in patients with PD compared to healthy controls. This decrease is even higher in neurons harboring α-Syn inclusions [156]. Besides, McNeil et al. (2014) suggested a clear negative correlation between the alpha-synuclein and LAMP-1 levels in experimental cell models. In their study, the authors demonstrated that ambroxol treatment significantly reduces α-Syn levels in neuroblastoma cells which is, in turn, accompanied by an increase in LAMP-1 levels [157]. 

### Autophagy Regulation in PD

As a crucial process for cell homeostasis and survival, autophagy is controlled at various levels. For instance, the forkhead-box O transcription factors are largely involved in the transcriptional control of autophagy [158]. FOXO3 directly binds to and regulates a complex of autophagy-related genes in adult neural stem cells [159]. It also stimulates FOXO1-mediated autophagy by activating the AKT1 signaling pathway [160]. Using a transcriptome-scale microarray approach, Dimitriu et al. (2012) discovered a significant upregulation of FOXO1 in the prefrontal cortex of PD patients. Moreover, a vast proportion of genes containing FOXO1-binding sites were also upregulated in the same brain area [161]. These findings are in agreement with an earlier microarray study by Zhang et al. (2005), in which FOXO1 showed consistent overexpression [162]. Intriguingly, the available expression data for another member of the family, FOXO3, are contradictory. Elevated activity and expression of FOXO3a has been closely associated with the Lewy bodies in the brain tissue of PD [163]. In contrast, significant downregulation of FOXO3 has also been described in PD brain samples [164]. Despite this inconsistency, the role of FOXO3 in neurodegeneration has been experimentally confirmed by functional analyses in transgenic cell models expressing wild-type, constitutively active, and dominant-negative variants of the gene. Further efforts are still needed for the better elucidation of FOXO3 involvement in PD development.

Another factor potentially implicated in autophagy regulation in PD is the inositol-requiring enzyme 1α/β (IRE1). IRE1 provides a direct link between protein accumulation and cell degeneration. Current evidence has suggested that IRE1 induces an autophagy-dependent neuronal death in an animal model of PD. Conversely, the suppression of IRE1 and ATG7 genes prevents the progression of α-Syn-induced PD in the same experimental model [165]. 

Mitophagy is a special case of (macro)autophagy that involves targeted degradation of mitochondria. Impaired mitochondrial clearance eventually leads to cell degeneration and death. The most extensively studied pathway for mitochondrial degradation requires PINK1/Parkin activation, even though this pathway is not the only mechanism of mitophagy [166,167,168,169,170]. Briefly, upon detection of dysfunctional mitochondria, PINK1 accumulates on the mitochondrial outer membrane where it activates Parkin. It in turn promotes the ubiquitination of mitochondria, marking them for clearance. Increased PINK1 expression exerts anti-apoptotic effects when cells are subjected to stress, while its deficiency makes cells prone to stress-induced cell death. The roles of PINK1 in mitochondria are versatile, including regulation of mitochondrial membrane potential, complex I and IV activity, ATP- and ROS- production. Comparably, the most remarkable feature of Parkin is its ability to provide cellular protection against a wide variety of detrimental factors, and its gene expression is upregulated in various types of stress [171]. Mutations in PINK1 and Parkin genes have been commonly associated with recessive forms of familial PD [172]. Loss of function of either of them results in impaired mitochondria accumulation. In addition, post-translational modifications of Parkin have proven important for its solubility or aggregation, and its potential contribution to the formation of Lewy bodies. 

## 4. The Complex Interplay between Autophagy and Neuroinflammation

The interaction between autophagy and neuroinflammation is complex and, to a certain extent, elusive.

A study involving murine models of PD has suggested that impaired autophagy enhances neuroinflammation in a NLRP3 inflammasome-dependent manner, eventually causing motor and cognitive impairments [173]. Conversely, artificially stimulated autophagy with rapamycin promotes a significant protective role not only in neurodegeneration but also in aging [174]. It has been also demonstrated that autophagy plays a crucial role in microglial activation in vitro. Suppression of microglial autophagy results in increased M1 microglial activation, leading to upregulation of pro-inflammatory cytokines [175].

Jin et al. (2018) revealed that TNF-α causes autophagy dysregulation in both neurons and microglia, which correlates with significant LAMP1 and LAMP2 overexpression in the microglia [176]. It has been suggested that the impaired autophagic flux stimulates microglial differentiation toward the M1 phenotype. In general, the M1 state of microglia is known as pro-inflammatory and neurotoxic, while M2 is anti-inflammatory and neuroprotective [177]. Therefore, impaired microglial autophagy may promote cell sensitization and neuroinflammation.

The importance of microglial autophagy for the efficient degradation of inflammatory myelin debris has been proven as a key mechanism in the control of the brain tissue homeostasis [178]. Autophagy-related phagocytosis improves the recovery rate of neuroinflammation and minimizes the symptoms of experimental autoimmune encephalomyelitis in murine models. 

Alterations in the gut microbiome and gut inflammatory processes are intimately linked to PD pathophysiology [179] and may have some bearing on the interactions of YKL-40, glia and autophagy, as highlighted above. Gut microbiome-derived butyrate, as a histone-deacetylase inhibitor, would be expected to regulate the capacity of NF-kB to induce YKL-40, as shown in glioma cells [180]. Butyrate’s mitochondria-optimizing effects seem to be at least partly dependent on its induction of sirtuin-3 to upregulate the mitochondrial melatonergic pathway, indicating diverse effects of butyrate arising from alterations in the availability of this pathway [181]. As melatonin suppresses LPS-induced YKL-40 [182] and acts in an autocrine manner to suppress glia activation, alterations in the availability of local melatonin and its capacity to be regulated by butyrate will be important to determine across cell types in PD patients and preclinical models.

## 5. Conclusions

Current diagnosis of PD relies almost entirely on evaluating the clinical presentations of the disease. Thus, the use of biomarkers that precisely indicate the stage and severity of the disease may improve diagnosis and facilitate therapeutic intervention. A large body of evidence has confirmed that autophagy and neuroinflammation are critical for PD development. These processes are regulated in a complex interconnected manner involving most of the known PD-associated genes. The extensive scientific efforts to clarify the molecular mechanisms of neuroinflammation and autophagy offer promising perspectives for the development of novel biomarkers that may objectively reflect the disease stage and severity. Furthermore, a better elucidation of the molecular basis of neuroinflammation and autophagy may allow these processes to be more precisely targeted by novel treatment strategies for PD. Probably the most valuable future approaches will integrate clinical observations with genetic and molecular data. Further efforts are still required for the complete elucidation of autophagy and neuroinflammation in the context of PD progression and therapy.
